# A Methodology Based on FT-IR Data Combined with Random Forest Model to Generate *Spectralprints* for the Characterization of High-Quality Vinegars

**DOI:** 10.3390/foods10061411

**Published:** 2021-06-18

**Authors:** José Luis P. Calle, Marta Ferreiro-González, Ana Ruiz-Rodríguez, Gerardo F. Barbero, José Á. Álvarez, Miguel Palma, Jesús Ayuso

**Affiliations:** 1 Department of Analytical Chemistry, Faculty of Sciences, University of Cadiz, Agrifood Campus of International Excellence (ceiA3), IVAGRO, 11510 Puerto Real, Spain; joseluis.perezcalle@uca.es (J.L.P.C.); ana.ruiz@uca.es (A.R.-R.); gerardo.fernandez@uca.es (G.F.B.); miguel.palma@uca.es (M.P.); 2 Department of Physical Chemistry, Faculty of Sciences, Institute of Biomolecules (INBIO), University of Cadiz, 11510 Puerto Real, Spain; joseangel.alvarez@uca.es (J.Á.Á.); jesus.ayuso@uca.es (J.A.)

**Keywords:** characterization, Fourier-transform infrared spectroscopy, cluster analysis, Sherry vinegar, spectralprint, random forest, support vector machine

## Abstract

Sherry wine vinegar is a Spanish gourmet product under Protected Designation of Origin (PDO). Before a vinegar can be labeled as Sherry vinegar, the product must meet certain requirements as established by its PDO, which, in this case, means that it has been produced following the traditional solera and criadera ageing system. The quality of the vinegar is determined by many factors such as the raw material, the acetification process or the aging system. For this reason, mainly producers, but also consumers, would benefit from the employment of effective analytical tools that allow precisely determining the origin and quality of vinegar. In the present study, a total of 48 Sherry vinegar samples manufactured from three different starting wines (Palomino Fino, Moscatel, and Pedro Ximénez wine) were analyzed by Fourier-transform infrared (FT-IR) spectroscopy. The spectroscopic data were combined with unsupervised exploratory techniques such as hierarchical cluster analysis (HCA) and principal component analysis (PCA), as well as other nonparametric supervised techniques, namely, support vector machine (SVM) and random forest (RF), for the characterization of the samples. The HCA and PCA results present a clear grouping trend of the vinegar samples according to their raw materials. SVM in combination with leave-one-out cross-validation (LOOCV) successfully classified 100% of the samples, according to the type of wine used for their production. The RF method allowed selecting the most important variables to develop the characteristic fingerprint (“spectralprint”) of the vinegar samples according to their starting wine. Furthermore, the RF model reached 100% accuracy for both LOOCV and out-of-bag (OOB) sets.

## 1. Introduction

The production of high-quality vinegar is increasingly important for manufacturers as consumers’ demand for a high-quality product presents a growing trend. The quality of vinegar is heavily determined by numerous factors, among which it is worth noting the raw material, the acetification system, and, in some cases, the specific wooden casks used for its aging [1].

In order to preserve and guarantee the quality of certain vinegars associated with specific geographical areas, the European Union recognizes these vinegars with the category of Protected Designation of Origin (PDO) (Council Regulation (EC) 510/2006). Such is the case of Sherry wine vinegar, from the Jerez-Xérès-Sherry, Manzanilla de Sanlúcar, and Vinagre de Jerez PDO region (in SW Spain). The quality of this PDO vinegar is related to the raw material (i.e., the grape variety), the production process, the type of cask used (America oak barrels), and the aging method [2]. This gourmet-grade wine vinegar is produced from high-quality Sherry wines which are, in turn, protected by a PDO that establishes very specific and traditional aging methods [3]. Both production and quality are precisely described and strictly regulated by law [4]. Sherry vinegar elaboration consists mainly of two production steps. The first step consists of the acetification procedure, which can be performed by traditional (oak barrels for several months) or industrial methods (steel tanks in just a few hours). Regardless of the acetification procedure applied, vinegar is in every case subjected to aging in oak barrels as the second production step. According to European Regulations, there are three categories of PDO Sherry vinegars depending on their aging time in oak wood barrels as follows: Vinagre de Jerez (at least 6 months of aging time), Vinagre de Jerez Reserva (at least 2 years of aging time), and Vinagre de Jerez Gran Reserva (at least 10 years of aging time) [5]. Vinegar diversity, increasing demand, and the fact that a convincing and objective authentication method is still an unresolved issue, the development of reliable analytical methods that allow establishing valid criteria regarding quality, origin, and verification of the production processes, such as aging, are required.

A large number of regular analytical methods have been developed until now for the characterization of different vinegar types on the basis of determining some of the individual compounds of interest that can be found in vinegar.

Since its aroma profile is considered one of the most important quality indicators of a particular vinegar, gas chromatography/mass spectrometry (GC–MS) continues to be the most widely employed technique for vinegar characterization and quality control [6]. In this sense, Pizarro et al. [7] characterized the volatile content in a number of vinegars to differentiate them according to raw material and production process (with or without aging in wood). Likewise, Marrufo-Curtido et al. employed stir bar sorptive extraction coupled to GC–MS (SPME–GC–MS) to characterize different vinegar samples—including Sherry vinegars—on the basis of the identification of certain volatile compounds, and they succeeded in classifying them by raw material and aging process [5]. Cejudo-Bastante et al. [8] performed a comparative study on the production of vinegar according to the acetification process used. For that purpose, they determined the vinegar samples’ polyphenolic and volatile profiles by gas and liquid chromatographic techniques. Ríos-Reina et al. compared three different sampling methods prior to analysis by GC–MS with the aim of determining the more suitable method for the characterization and differentiation between vinegar PDOs and other categories [6].

Other methods such as inductively coupled plasma optical emission spectroscopy (ICP-OES) were also successfully used to characterize the mineral composition of a number of PDO Andalusian wine vinegars and classify them according to their origin [9]. Furthermore, ^1^H-NMR combined with pattern recognition analysis was successfully used to classify vinegars and wines from different raw materials [10,11].

Although all these methods achieved good results, they also present some drawbacks, since they all require costly equipment, long analysis time, and highly qualified personnel. Although such methods can be considered as perfectly suitable for research purposes, they are deemed otherwise within a regular winery environment. Consequently, spectroscopic methods are becoming more popular for the control of vinegar processes at an industrial scale, since they are rapid, nondestructive, and easily applied in situ. In addition, they require minimal or hardly any sample preparation. These attributes make spectroscopic techniques a more appropriate option for the development of vanguard/rearguard analytical strategies for the control of production processes, so that the need for specific corrective actions can be determined in the shortest possible time [12]. Numerous methods can be found in the literature where the individual identification of the compounds of interest in wine, as well as in related drinks, is conducted by Fourier-transform infrared spectroscopy (FT-IR) [13,14]. It must be noted that the IR spectra from wines, vinegar, and other beverages are complex mixtures of overlapped peaks, i.e., unresolved peaks. Therefore, they cannot be used as regular spectroscopic methods; instead, the application of multivariate regression techniques is mandatory. Individual compounds can be determined; however, no specific signals are used. Instead, a model including many different signals must be used.

However, although most of these spectroscopic methods are based on the identification of individual chemical compounds, in order to ensure vinegar authenticity, several compounds would have to be quantified. On the other hand, more sophisticated fake production methods are being continuously developed, which means that certain minor variations that can make a substantial difference in certain cases represent an increasingly demanding challenge. Thus, the characterization of samples based on a limited number of markers can be sometimes complicated and time-consuming. For this reason, nontargeted chemical analyses based on spectroscopic techniques combined with chemometrics are becoming more frequently used for food characterization [15,16]. In this sense, the use of the whole spectral range in combination with chemometrics to identify a unique fingerprint, which has been recently given the name of spectralprint, allows a rapid characterization of each sample with minimum or no preparation at all [13]. In this sense, Ríos-Reina et al. applied fluorescence excitation–emission spectroscopy coupled to parallel factor analysis (PARAFAC) and support vector machine (SVM) to characterize and classify the three abovementioned Spanish wine vinegars with PDO and concluded that SVM classification models provide higher predictive accuracy (over 92%) [17]. The same authors demonstrated the effectiveness of other spectroscopic techniques, namely, near-infrared spectroscopy (NIRS) in combination with certain chemometrics such as principal component analysis (PCA) and partial least squares - discriminant analysis regression (PLS-DA) regarding a rapid and reliable classification and authentication of Spanish PDO wine vinegars [18]. However, to date, only a few papers have been published regarding the use of FT-IR in combination with pattern recognition techniques to discriminate wine vinegars according to their origins. In this sense, the capacity of FT-IR in combination with PLS for the characterization of Sherry wine according to its aging process was previously studied [19]. Guerrero et al. proved that mid-IR spectroscopy combined with multivariate chemometric techniques could be successfully used to classify vinegar samples elaborated from different raw materials, including apple, white/red wines, or balsamic vinegars, as well as their production processes (with and without aging in wood), with 89% accuracy [20]. Ríos-Reina et al. also studied the potential of FT-IR for the characterization of vinegar and classified them according to the standardized aging categories of high-quality wine vinegars [2]. A reduced number of studies can be found in the literature that investigated the use of the whole ultraviolet/visible (UV/Vis) spectra for vinegar authentication [21].

According to the recently published review by Ríos-Reina et al., there are not many studies where the suitability of the different spectroscopic techniques that have been used in this field were compared for effectiveness [13]. Thus, the development of an analytical method that is suitable to be implemented as a routine for the characterization of vinegar remains a challenge. The same review reported that parametric techniques such as PCA, partial least squares (PLS), PLS-DA, and linear discriminant analysis (LDA) are the most often used chemometric tools. However, the implementation of nonparametric techniques such as SVM or, more specifically, random forest (RF) to the characterization of wine or vinegar is scarce or even nonexistent. Nevertheless, according to several studies with a similar approach, the use of nonparametric techniques has been reported to provide better results [22,23].

Therefore, the aim of this work was to design an analytical method based on FT-IR spectral profiles in combination with support vector machine and random forest to produce *spectralprints* that allow the classification of Sherry wine vinegar according to their starting wine.

## 2. Materials and Methods

### 2.1. Samples

A total of 48 samples obtained from different Sherry vinegars under Protected Designation of Origin (PDO) from a local winery (Bodegas Páez Morilla S.A., Jerez de la Frontera, Spain). All of the samples were “Reserva” vinegars, i.e., more than 2 years of aging in oak barrels. The samples were taken directly from certain oak barrels in the winery. The vinegar samples were divided into three categories accordingly to the origin of the wine used to elaborate the vinegar as follows: 24 Palomino Fino vinegar, 12 Moscatel vinegar, and 12 Pedro Ximénez vinegar. In order to ensure a wide assortment, the samples from the winery were taken directly from oak barrels of varied volume located inside different buildings, at different positions, and containing different starting grape/wines. The samples were tagged after their specific starting wine as follows: PF for Palomino Fino, MO for Moscatel, and PX for Pedro Ximénez, followed by the barrel row level indicated by OB1 for solera (ground level) and OB2 for first criadera (first level)—corresponding to different aging times. In order to reduce turbidity and remove impurities, before being subjected to FT-IR spectrophotometry, the samples were filtered through 0.45 µm filters. No further preparation procedures were required. In addition, all samples were analyzed in duplicate, and the average value for each sample has been used.

### 2.2. Fourier-Transform Infrared Spectra Acquisition

Fourier-transform infrared spectra were obtained for all samples by means of a MultiSpec (TDI, Barcelona, Spain) spectrophotometer. A 7 mL (standard setting) sample was collected and pumped through the system. The spectra were recorded in the range 952–3070 cm^−1^ with 3.86 cm^−1^ resolution and 20 μm optical path length. The operating temperature was maintained at 25 °C. The total process time per sample was 1 min. All of the samples were measured after doing a blank using a commercial solution provided by TDI, which is a water solution of Triton^®^ (TDI, Barcelona, Spain).

### 2.3. Data Analysis

No data pretreatment was performed. Thus, the spectral raw data were placed into D_nxp_ matrices where *n* denotes the number of samples and *p* denotes the number of variables. Therefore, a D_48×555_ (48 spectra recorded at 555 different wavenumbers) matrix was obtained for multivariate analysis. The processing of the data, using both unsupervised techniques such as hierarchical cluster analysis (HCA) and supervised nonparametric techniques such as support vector machine (SVM) or random forest (RF), was performed using RStudio software (R version 4.0.5, Boston, MA, USA).

## 3. Results

### 3.1. Exploratory Analysis

**Figure 1:**
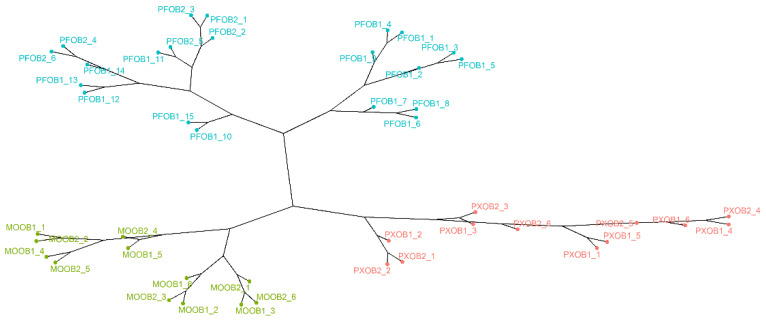
Dendrogram from the HCA analysis combined with Ward’s method with Manhattan distance. The vinegar samples are colored according to their starting wine: blue for PF (Palomino Fino), green for MO (Moscatel), and pink for PX (Pedro Ximénez). OB1: solera and OB2: first criadera (*n* = 48).

Firstly, each sample’s (*n* = 48) raw FT-IR spectrum without any pretreatment (*p* = 555) was subjected to hierarchical cluster analysis (HCA) followed by Ward’s method with Manhattan distance to determine any clustering trends. The resulting dendrogram, represented as a phylogenetic tree for easier comprehension, can be seen in Figure 1.

As can be seen, three clearly differentiated branches were obtained—each one corresponding to each of the three starting wines used to elaborate the vinegars. It can be observed that the PX samples (pink color) were closer to the branch containing the MO samples (green color). This suggests that these two types of wine vinegars have a closer similarity with regard to their FT-IR spectrum. Additionally, Figure S1 (Supplementary Materials) shows the FT-IR spectra for all of the vinegar samples, where this greater similarity can be observed. On the other hand, the PF samples (blue color) were the most clearly differentiated from the rest of the samples, especially from the PX ones, which were farther apart. These trends could be associated with the specific ethanol initial content in each starting wine, since PF wines generally exhibit lower ethanol content than MO wines and substantially lower content than PX wines. It must be mentioned that the total acidity of all the vinegars analyzed in this study did not significantly fluctuate.

It should also be noted that the PF group was the most heterogeneous and that it could be divided into two different sub-branches: one exclusively including samples aged for longer times, i.e., PFOB1, and the other comprising all of the PFOB2 samples plus the remaining PFOB1 samples. These results may suggest that FT-IR could also be somewhat correlated with aging time, even if a clear trend in this sense was not ascertained.

It could be said that, in general, this unsupervised exploratory analysis brought to light some data patterns that would allow differentiating between wine vinegars regardless of their aging time.

**Figure 2:**
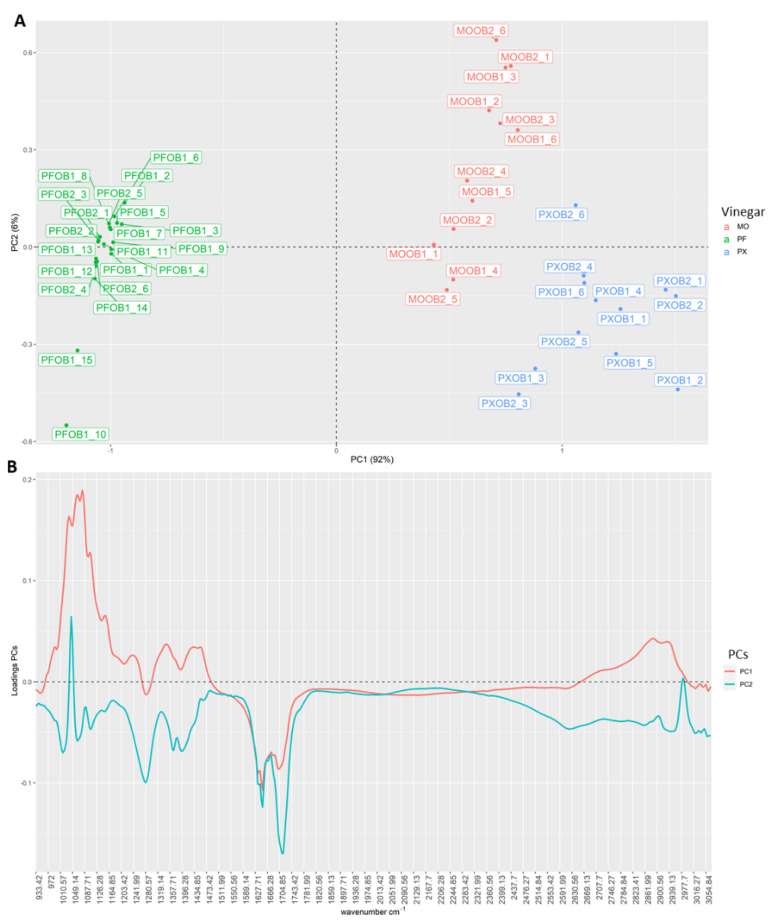
(**A**) Scores obtained by all the samples for the first two principal components (PC1 and PC2); (**B**) loadings obtained in each of the PCs.

Additionally, to corroborate this clustering pattern, as well as the wavenumbers responsible for this trend, principal component analysis (PCA) was carried out. Figure 2A shows the scores obtained by the observations for PC1 and PC2. Figure 2B represents the loadings obtained in each of the PCs. As can be seen, in Figure 2A, the grouping trend was exactly the same as in HCA. In this case, PC1 (explaining 92% of the variability of the data) was mainly responsible for the separation of the samples according to the starting wine vinegar. Thus, PF samples were farther away from the rest, acquiring negative scores for PC1, while MO and PF samples were closer to each other with positive scores. However, the three groups were clearly differentiated. Figure 2B gives an idea of the most important wavenumbers for such a separation. The highest loadings were obtained for the region from 972 to 1174 cm^−1^, which is related to hydroxyl group (C–O stretching of alcohol). In addition, other spectral regions such as 1600 cm^−1^ (related to aromatic compounds) and 2850 cm^−1^ (related to the O–H stretching of acid components) seem to be important according to the PCA results.

### 3.2. Supervised Techniques

Both HCA and PCA analyses achieved a quite thorough separation of the vinegar samples according to the type of starting wine. However, this technique does not allow predicting future observations; thus, it is necessary to elaborate a predictive model. For our study, support vector machine and random forest were selected as nonparametric techniques that would allow a predictive model to be generated. According to the most recent studies, RF has never been applied to wine or vinegar samples, while SVM has rarely been used for this purpose [13]. However, both models have exhibited considerable potential when applied to other foodstuffs [22,23].

#### 3.2.1. Support Vector Machine (SVM)

**Figure 3:**
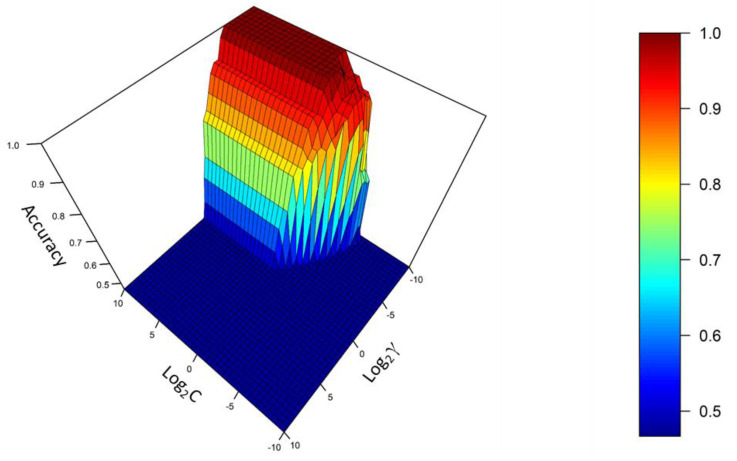
Accuracy of the SVM model calculated using k-fold cross-validation according to log_2_C and log_2_γ values.

Support Vector Machine is a supervised method that is commonly used for classification purposes. It is based on a concept known as hyperplane. Hyperplanes allow a clear separation of the items observed according to support vectors. Thus, there is a hyperparameter known as cost (C) that controls the number of support vectors and, consequently, the balance between bias and variance. Furthermore, for this type of analysis, a Radial basis function (RBF) is used so that the separation limits are not linear. Thus, a new hyperparameter known as gamma (γ) is introduced to control the behavior of the Gaussian kernel. Both hyperparameters are to be determined by the analyst and, for this purpose, fivefold cross-validation was used [24,25]. In this case, a grid search method where sequences of C and γ grow exponentially was chosen. Thus, the values in the range (−10, 10) with an increment step of 0.5 units were taken for log_2_C and log_2_γ. Note that, for the fivefold cross-validation, the dataset was divided into five subsets of equal size. Four of such subsets were used to train the model, and the remaining one was used as a test. This process was repeated for each of the subsets. Thus, 8405 models were generated, i.e., 41 × 41 (combinations of C and γ) × 5 (subsets). Figure 3 represents the log_2_γ values (*y*-axis) versus the log_2_C values (*x*-axis) and the accuracy obtained (*z*-axis). As can be seen, for gamma values roughly below 0.031 (log_2_γ = −5) and cost values above 1 (log_2_C = 0), the accuracy stabilized at the maximum level. On the one hand, the best results were obtained with the lowest values of gamma and, since this hyperparameter controls the behavior of the kernel, this suggests that the groups were practically linearly separable. In this case, the optimal gamma value was established at 0.00781 (log_2_γ = −7). On the other hand, it seems that the accuracy increased with higher C values. Since this hyperparameter controls the balance between bias and variance, this indicates that fewer misclassified observations would be allowed by the hyperplane and, consequently, there would be fewer support vectors, resulting in a less biased model but with a higher variance. For this reason, the optimal value for C was established at 1 (log_2_C = 0), since it was the lowest value allowing maximum accuracy. In this way, overfitting (lower variance) was avoided, and excellent performance (lower bias) was achieved, corroborating the robustness of the predictive model. Using the abovementioned hyperparameters, a new model was trained and leave-one-out cross-validation (LOOCV) was performed to determine the error. Both the trained and the LOOCV sets exhibited 100% accuracy, confirming the good performance of the model.

Although the SVM model proved excellent behavior, the nature of the algorithm does not allow the selection of the most relevant variables regarding the definition of each vinegar *spectralprint* for classification purposes. Consequently, another nonparametric technique known as random forest (RF) was used for that purpose.

#### 3.2.2. Random Forest (RF)

**Figure 4:**
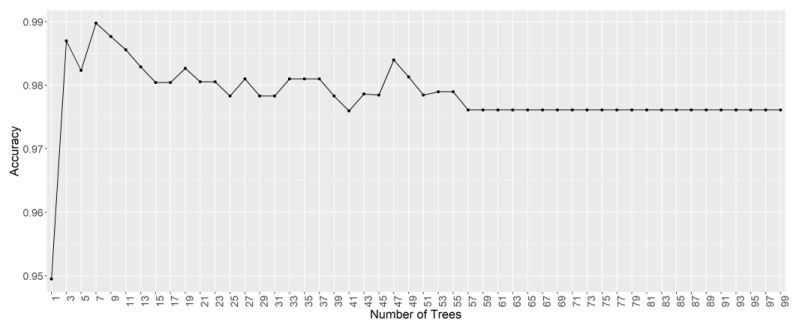
Accuracy of the RF model according to the number of trees.

Random forest is a nonparametric supervised technique commonly used for classification and regression purposes. The RF model is made up of multiple individual decision trees trained with a series of random training sets generated by bootstrapping (sampling with replacement). Therefore, there is a data subset, known as out of bag (OOB) which does not contribute to create the model. Thus, in order to evaluate the model performance, a cross-validation method of these OOB instances can be used to determine an unbiased generalization error [26]. Additionally, RF trees are decorrelated by randomly selecting m predictors before evaluating each split in an individual tree. The m value, known as mtry, is a hyperparameter to be optimized by the analyst. For classification purposes, the square root of the total number of predictors is generally set to 24. In addition, a specific number of trees in the RF model must be established. In this sense, a greater number of trees does not result in a greater risk of overfitting, although it should be noted that an excessive number of trees demands longer computation times. Thus, 23 was selected as the mtry value, i.e., the square root of the number of predictors (555 variables). In order to determine the number of trees to be used, models from 2–100 at two-tree intervals were created using the accuracy of the OOB dataset as the criterion (Figure 4). Although the error stabilized rather quickly (at approximately 57 trees), it was continued up to the 100-tree model. In this case, the model with 100 trees was chosen, since it was close to twice the number of trees where the error stabilized.

The model accuracy with the training set was 100%. In addition, two external validations were performed on the OOB and LOOCV sets, with accuracy levels at 97.24% and 100%, respectively. It was, therefore, confirmed that a highly reliable and accurate model was obtained.

Given the nature of the RF model, it is possible to select the most relevant variables for classification purposes. In this case, increasing node purity was the selection criterion.

The six most relevant wavenumbers selected in the RF included some signals related to C–O stretching (1099.28, 1145.57, and 1218.85 cm^−1^), C–H bending (1457.99 and 1469.56 cm^−1^), and O–H stretching in a carboxylic acid (2842.7 cm^−1^). An ANOVA was performed for each of the selected variables, and all of them were significant at a confidence level of 5%.

In order to verify that the model remained stable when based on just those data, a new RF model was elaborated using just those 6 variables. In this case, the value of mtry was set to 2 and the number of trees was set to 100. The result showed 100% accuracy for the training set, as well as for the OOB and LOOCV sets. A summary of the accuracy level achieved by this model (reduced RF) and by the model including all the variables (complete RF) is shown in Table 1. It is worth noting the higher accuracy obtained on the OOB dataset by the reduced RF. This could be explained by the random selection of the samples that make up the OOB set, resulting in hardly any differences between both models. The high collinearity between the predictors in the spectroscopic data is also worth noting. Such collinearity could be explained by the related contributions from the major compounds in the sample, i.e., acetic acid and ethanol, as well as other carboxylic acids and alcohols. Therefore, by reducing from 555 to six variables, this collinearity was reduced and, consequently, accuracy was expected to increase. The possible noise reduction was also expected to contribute to an improvement in the model accuracy. Nevertheless, given the minor error and that collinearity is not so relevant regarding RF models, both of them—complete and reduced models—were considered stable.

**Figure 5:**
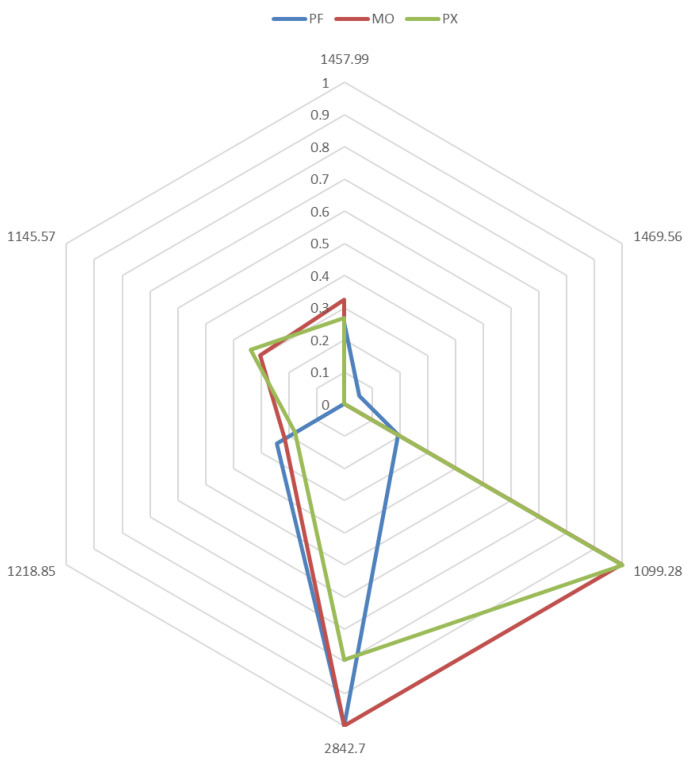
Characteristic *spectralprint* of each wine vinegar type.

Therefore, the selected variables were used to perform a *spectralprint* that can be used as a suitable routine method for the rapid, reliable, and straightforward identification of wine vinegar types. Figure 5 displays in a radial graph the characteristic *spectralprint* corresponding to each type of wine vinegar. In addition, the mean values of the six variables in each group were normalized to the base peak at 100%. Therefore, intensities and ratios could be used to clearly distinguish each type of wine vinegar. As can be seen, the fingerprint created by each type of vinegar was different.

PF vinegar, in particular, presented its maximum intensity at wavenumber 2842.7 cm^−1^ and remained below 0.3 at the remaining wavenumbers. This profile was completely different from that of the other groups, especially at wavenumbers 1099.28 cm^−1^ and 1145.57 cm^−1^. The MO and PX vinegar samples presented more similar profiles, as expected in view of the data from the HCA and PCA analyses, with very high intensities at wavenumber 1099.28 cm^−1^. However, even if their values at wavenumbers 2842.7, 1218.85, 1145.57, and 1457.99 cm^−1^ diverged, the most notable difference appeared at wavenumber 2842.7 cm^−1^, where MO vinegar showed its maximum intensity, while PX vinegar reached an intensity around 0.7. The remaining variables, as well as their ratios, were also different for each type of vinegar, thus giving different spectralprints that can be used for the discrimination of the vinegar samples based on the starting wine. This notable signal at 2842.7 cm^−1^ can be attributed to the O–H stretching in carboxylic acid, which suggests that it is similarly and directly related to both prefermentative and fermentative acids, i.e., tartaric, malic, and/or citric acids and succinic, lactic, and acetic acids respectively.

The specific wavenumbers in the spectrum are strongly related to certain specific components associated to the organoleptic properties of vinegar. Thus, some absorptions by hydroxyl groups (C–O stretching of alcohol) were specifically recorded between 1010 cm^−1^ and 1150 cm^−1^. These absorptions came from ethanol and fusel oils, including propyl alcohol, butyl alcohols, isoamyl alcohols, and hexanol—all of them associated with organoleptic properties. The wavenumbers 1099.28 and 1145.57 cm^−1^, which were previously identified as the most relevant values with regard to the discrimination of PF vinegars from the other wine vinegars, were within this region. The region between 2840 cm^−1^ and 2940 cm^−1^ presented several absorptions resulting from the O–H stretching of the acid components. The most relevant ones were organic acids such as acetic and tartaric, as well as citric, malic, succinic, and lactic acids. As already mentioned, wavenumber 2842.7 cm^−1^ within this range is quite relevant regarding the discrimination of both PX and MO vinegars. The region between 1450 cm^−1^ and 1510 cm^−1^ describes the absorptions related to aromatic rings (C=C–C stretching). The wavenumbers 1457.99 cm^−1^ and 1469.56 cm^−1^ in this region, which were selected to generate the *spectralprints*, are associated with many of the aromatic compounds in these vinegars. Lastly, the wavenumber 1218.85 cm^−1^ is related to C–O stretching in different kinds of compounds (region from 1200 to 1225 cm^−1^).

## 4. Conclusions

FT-IR combined with chemometric tools was demonstrated to be a quite practical methodology for the characterization of Sherry vinegars according to their origin. Specifically, the SVM algorithm applied to the vinegar samples achieved 100% accuracy of the LOOCV. The RF model also displayed an excellent performance at 100% LOOCV and 97.23% OOB accuracy. In addition, the six most relevant wavenumbers were selected from the RF model to create a new RF model that achieved 100% accuracy for both validations (LOOCV and OOB). This is the first time that the RF algorithm has been applied to wine and vinegar samples, and that its validity and robustness have been demonstrated. In addition, the six most relevant wavenumbers were also used to create a characteristic *spectralprint* for each type of wine vinegar, which allowed their rapid, reliable, and uncomplicated differentiation according to their starting wine.

To conclude, spectroscopic techniques were proven to be nondestructive and environmentally friendly methodologies with the capacity to provide rapid and highly reliable results regarding the characterization of vinegars. This, together with their simplicity of use, portability, and low-demanding investment, makes them a highly recommended methodology for in situ routine control of the production and aging processes of vinegars in wineries. In addition, a web platform can be developed with the generated models in order to facilitate data analysis for other users, making the characterization process even easier and more automated.

## Figures and Tables

**Table 1:** Accuracy of both RF models for the different validations.

	Accuracy (%)		
	Training Set	OOB Set	LOOCV
Complete RF	100	97.24	100
Reduced RF	100	100	100

## Data Availability

The data presented in this study are contained within the article.

## Supplementary Materials

The following are available online at https://www.mdpi.com/article/10.3390/foods10061411/s1: Figure S1. FT-IR spectrum of all of the samples (D_48×555_). Samples are colored according to the type of wine vinegar: MO (pink), PF (green), and PX (blue).
