# Supplementary material for: A Methodology Based on FT-IR Data Combined with Random Forest Model to Generate Spectralprints for the Characterization of High-Quality Vinegars
Source: Foods. 2021 Jun 18;10(6):1411. doi: 10.3390/foods10061411 (PMC8233915; doi:10.3390/foods10061411)
Supplement: Supplementary file 1 [file foods-10-01411-s001.zip › foods-1250387-supplementary.pdf]

foods

MDPI

Article

A Methodology Based on FT-IR Data Combined with Support Vector Machine and Random Forest Models to Generate *Spectralprints* for the Characterization of High Quality Vinegars

José Luis P. Calle [1], Marta Ferreiro-González [1,*], Ana Ruiz-Rodríguez [1], Gerardo F. Barbero [1], José Á. Álvarez [2], Miguel Palma [1] and Jesús Ayuso [2]

[1] Department of Analytical Chemistry, Faculty of Sciences, University of Cadiz, Agrifood Campus of International Excellence (ceiA3), IVAGRO, 11510 Puerto Real, Cadiz, Spain; joseluis.perezcalle@uca.es (J.L.P.C.); marta.ferreiro@uca.es (M.F.-G.); ana.ruiz@uca.es (A.R.-R.); gerardo.fernandez@uca.es (G.F.B.); miguel.palma@uca.es (M.P.)

[2] Department of Physical Chemistry, Faculty of Sciences, Institute of Biomolecules (INBIO), University of Cadiz, 11510 Puerto Real, Cadiz, Spain; joseangel.alvarez@uca.es (J.Á.Á.); jesus.ayuso@uca.es (J.A.)

* Correspondence: marta.ferreiro@uca.es; Tel.: +34-956-01-6359

Supplementary Materials:

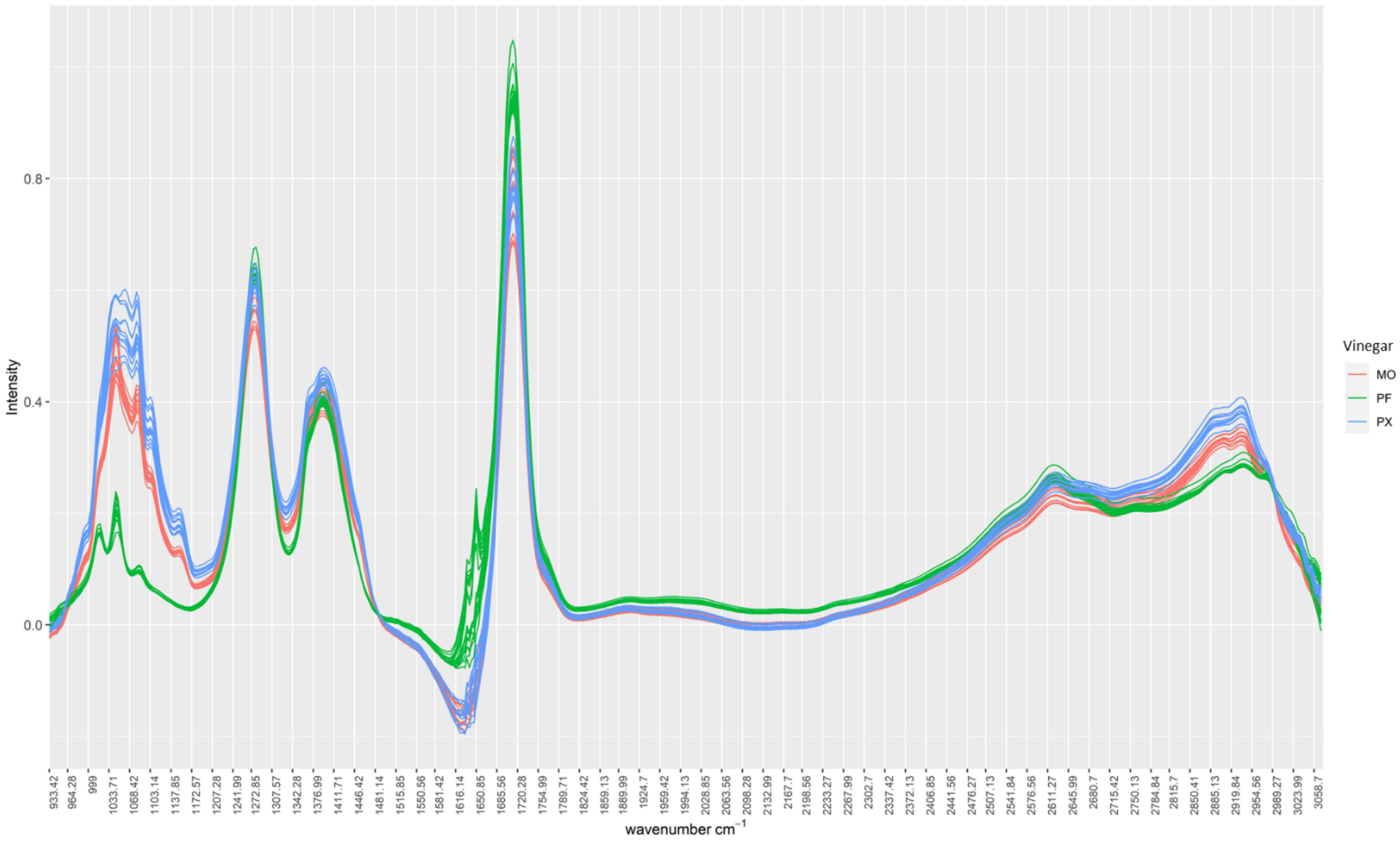

Figure S1. FT-IR spectrum of all of the samples (D_{48x555}). Samples are colored according to the type of wine vinegar: MO (pink), PF (green) and PX (blue).
